# FRAILTY IN MIDLIFE AS A PREDICTOR OF CHANGES IN BODY COMPOSITION FROM MIDLIFE INTO OLD AGE

**DOI:** 10.1093/geroni/igad104.3361

**Published:** 2023-12-21

**Authors:** Laura Kananen, Markus Haapanen, Tuija Mikkola, Juulia Jylhävä, Niko Wasenius, Johan Eriksson, Mikaela von Bonsdorff

**Affiliations:** Tampere University, Tampere, Pirkanmaa, Finland; University of Helsinki, Helsinki, Uusimaa, Finland; Folkhälsan, Helsinki, Uusimaa, Finland; Karolinska institute, Stockholm, Stockholms Lan, Sweden; University of Helsinki, Helsinki, Uusimaa, Finland; University of Helsinki, Helsinki, Uusimaa, Finland; University of Jyväskylä, Jyväskylä, Keski-Suomi, Finland

## Abstract

Few studies have investigated the association between frailty and subsequent body composition. We performed separate linear mixed model analyses in 996 adults (mean age at baseline[SD]: 61.5[2.9]) in a Finnish longitudinal birth cohort study to explore the relationships between changes in frailty status assessed by the Rockwood 41-item-frailty-index (FI) and changes in body mass index (BMI), lean mass index (LMI), fat mass index (FMI), and FMI to LMI-ratio values during 17 years of follow-up. With advancing age, LMI and BMI decreased, whereas FMI and FMI to LMI -ratio increased. Those who were frail (FI≥ 0.25) already at baseline, followed by those who became frail during the follow-up, experienced faster decreases in LMI and faster increases in FMI and FMI to LMI -ratio values relative to those who were ‘never frail’. Contrastingly, those in the highest third of absolute annual increase in FMI and FMI to LMI-ratio became frailer faster over time relative to those in the lowest third. We found evidence of an adverse health outcome of frailty where lean indices declined faster and fat indices and fat to lean -ratios increased faster from midlife into old age. The changes resembled those that occurred with aging, but at a faster pace. The relationship between body composition and frailty is likely bidirectional, where high or increasing levels of fat are associated with the risk of becoming frailer earlier, but where a longer duration of frailty may increase the risk of faster age-related changes to body composition.

